# Body mass index is not a reliable tool in predicting celiac disease in children

**DOI:** 10.1186/1471-2431-14-165

**Published:** 2014-06-30

**Authors:** Maria van der Pals, Anna Myléus, Fredrik Norström, Solveig Hammarroth, Lotta Högberg, Anna Rosén, Anneli Ivarsson, Annelie Carlsson

**Affiliations:** 1 Department of Pediatrics, Clinical Sciences, Skåne University Hospital, Malmö, Lund University, SE-205 02 Lund, Sweden; 2 Department of Public Health and Clinical Medicine, Epidemiology and Global Health, Umeå University, Umeå, Sweden; 3 Pediatric Clinic, Norrtälje Hospital, Norrtälje, Sweden; 4 Division of Pediatrics, Department of Clinical and Experimental Medicine, Faculty of Health Sciences, Linköping University, Department of Pediatrics in Norrköping, County Council of Östergötland, Norrköping, Sweden

**Keywords:** Body mass index, Celiac disease, Children, Height, Screening study, Weight

## Abstract

**Background:**

Untreated celiac disease is traditionally believed to be associated with malabsorption and underweight. However, studies describing body mass index (BMI) in individuals at the time of diagnosis have shown contradictory results. We investigated the differences in weight, height, and BMI in 12- year-old children with screening-detected celiac disease compared to their healthy peers.

**Methods:**

In a population-based screening study of 12,632 12-year-old children, blood samples were analyzed for markers of celiac disease. Children with elevated markers were referred for a small bowel biopsy. Weight and height were measured in 239 out of 242 children with screening-detected celiac disease (57.3% girls) and in 12,227 children without celiac disease (48.5% girls). BMI was categorized according to the International Obesity Task Force. Age- and sex-specific cut-off points for underweight, normal weight, and overweight were used.

**Results:**

Children with celiac disease weighed less and were shorter than their peers (median weight 45.2 kg, interquartile range (IQR) 40.2–52.2 kg vs. 47.0 kg, IQR 41.1–54.4 kg, respectively, p = 0.01; median height 156.5 cm, IQR 151.0–162.0 cm vs. 157.5 cm, IQR 152.0–163.0 cm, respectively, p = 0.04). In comparing those with celiac disease to their healthy peers, 4.2% vs. 5.2% were underweight, 82.0% vs. 72.8% were normal weight, and 13.8% vs. 21.9% were overweight, respectively. There was no association between being underweight and the risk of having undiagnosed celiac disease (Odds ratio (OR) 1.3, 95% CI 0.7–2.4), but the risk was significantly lower among overweight children (OR 0.56, 95% CI 0.4–0.8). Median BMI was slightly lower among the children with screening-detected celiac disease compared to their healthy peers (18.6 kg/m^2^, IQR 17.1–19.8 kg/m^2^ vs. 18.8 kg/m^2^, IQR 17.2–21.1 kg/m^2^, respectively, p = 0.05), but most of the celiac disease cases had a normal BMI.

**Conclusions:**

At a population level, children with celiac disease weigh less, are shorter, and have a lower BMI compared to their peers without celiac disease, and this emphasizes the importance of early recognition and treatment of the condition. However, at an individual level, growth parameters are not reliable in establishing the diagnosis.

## Background

Celiac disease is one of the most common chronic diseases in childhood and affects approximately 0.5%–3% of the population in the Western world [1-3]. It is characterized by an autoimmune response triggered by gluten and other environmental cofactors that leads to small-intestinal mucosal injury [4]. The disease can have its onset at any age throughout life, and its clinical expression is heterogeneous. The classic presentation of celiac disease is commonly described as diarrhea, abdominal distention, malnutrition, and failure to thrive [5-7]. Younger children often present with gastrointestinal symptoms and weight loss, but the clinical presentation seems to have changed in recent decades and the proportions of patients suffering from classical gastrointestinal symptoms, including weight loss, are decreasing. More patients now suffer from extra-intestinal symptoms or have no symptoms and are found in screening studies [8,9]. In contrast to previous beliefs, it is now well established that many adult patients with celiac disease have a high or normal body mass index (BMI) at diagnosis [10-13]. Some studies show that BMI increases on a gluten-free diet, especially in those who adhere closely to the diet [14]. However, other studies describing BMI in individuals at diagnosis of celiac disease and/or after introduction of a gluten-free diet have shown contradictory results. Few studies examining BMI and other growth parameters have been performed in children, and the findings of those studies have been inconclusive [15-17].

The main objective of this study was to examine weight, height, and BMI in 12-year-old children with untreated screening-detected celiac disease and to compare these parameters with their healthy peers.

## Methods

### Study design

The present investigation was based on the children included in the ETICS study (Exploring the Iceberg of Celiacs in Sweden). Details of the celiac disease screening strategy and descriptions of the included children have been published previously [3,18]. In brief, ETICS is a school-based cross-sectional multicenter national screening study for celiac disease in 12-year-old children and is part of the Prevent-CD European project [19]. Participating families gave their signed informed consent before the children were enrolled. Children with an existing diagnosis of celiac disease (n = 96) were excluded from this study. Blood samples from all children were analyzed for anti-human tissue transglutaminase (tTG) and, if borderline values were obtained, also for endomysial antibodies. Children with values above a predefined cut-off were referred to the closest pediatric clinic for a small-intestinal biopsy [20,21]. Criteria for diagnosis were Marsh 3a-c enteropathy or a combination of Marsh 1 and Marsh 2 enteropathy, HLA-DQ2/DQ8 haplotype, and symptoms and/or signs compatible with celiac disease [21]. Genotyping for HLA alleles encoding for HLA-DQ2/DQ8 was performed by oligonucleotide probe hybridization and was verified in all screening-detected cases. The study was approved by the Regional Ethical Review Board of Umeå University, Umeå, Sweden.

### Anthropometric assessment

Weight and height were measured at the time of the screening for celiac disease according to standard procedures. All school nurses were given uniform instructions on how to carry out these measurements. The scales were all recently calibrated, and a wall-mounted stadiometer was used for measuring height. The children wore light clothing and no shoes and were measured with their body in a straight line and their head in an appropriate position. BMI was calculated as weight (kg) divided by the square of the height (m^2^) and categorized using the cut-off points recommended by the International Obesity Task Force (IOTF) [22]. Age- and sex-specific cut-off points corresponding to the adult BMI value of <18.5 (defined as underweight) and ≥ 25 (defined as overweight) were used. As a reference, adult BMI 25 corresponds to 21.22 for 12-year-old boys and to 21.68 for 12-year-old girls and adult BMI 18.5 corresponds to 15.35 for 12-year-old boys and to 15.62 for 12-year-old girls [22,23].

### Statistical analysis

Microsoft Access 2010 (Microsoft, Redmond, WA) was used for handling the ETICS database, and statistical analysis was performed using SPSS Statistics for Windows (Version 21.0, IBM Corp, Armonk, NY). Continuous variables are reported as the median and interquartile range (IQR) because of unequal sample size and skewed distributions. The 25th and 75th percentiles are described in the text. Categorical variables are reported as the number and percentage of subjects with the characteristic of interest. Between-group comparisons were performed with the Wilcoxon–Mann–Whitney test for continuous variables and with chi-square or Fisher’s exact test for categorical variables as appropriate. These tests were performed on the whole group as well as after stratifications. Univariate logistic regression models tested the odds of having celiac disease while being underweight or being overweight. Statistical significance was defined as a two-sided P ≤ 0.05 or an odds ratio (OR) with a 95% confidence interval (CI) not including 1.

## Results

**Figure 1:**
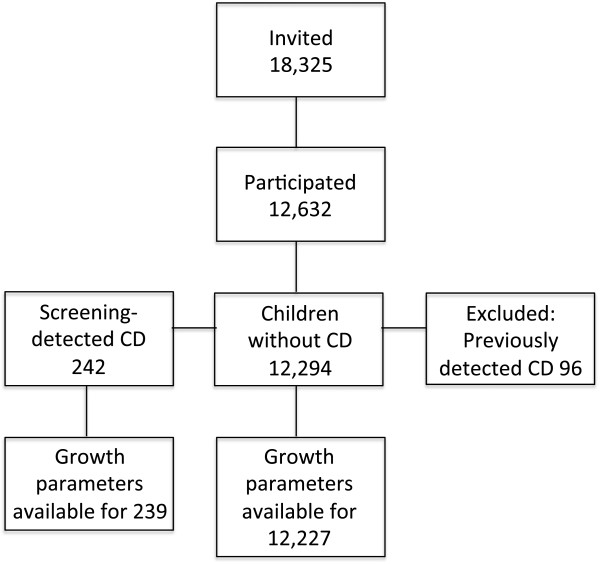
**Flowchart depicting the screening procedure.** Cross-sectional screenings performed in 12-year-old children across Sweden to investigate the prevalence of celiac disease (CD) and to assess the growth parameters in comparison with healthy children. The numbers of children are given in the boxes.

In total, 12,632 children (69% of those invited) participated. Details regarding the prevalence of celiac disease have been published previously [3,18]. In total, 242 newly detected celiac disease cases were identified within the study as a result of the screening. Weight and height were available in 239 (99%) children with newly detected celiac disease (57.3% girls) and in 12,227 (99%) of the study participants without celiac disease (48.5% girls) (Figure 1).

The children with screening-detected celiac disease weighed less on average compared to the children without celiac disease (median weight 45.2 kg, IQR 41.1–54.4 kg vs. 47.0 kg, IQR 40.2–52.2 kg, p = 0.01) (Table 1). There was no statistically significant difference in weight between girls and boys within the celiac disease group (p = 0.86). The children with screening-detected celiac disease were also significantly shorter compared to the children without celiac disease (median height 156.5 cm, IQR 151.0–162.0 cm vs. 157.5 cm, IQR 152.0–163.0 cm, p = 0.04).

**Table 1:** Comparison of BMI, weight and height between children without celiac disease (CD) and children with screening-detected celiac disease

**Groups, n (%)**	**No CD n = 12,227**	**CD n = 239**	**P-value**^**a**^
Girls, n (%)	5932 (48.5)	137 (57.3)	
Age-and sex-adjusted BMI, n (%)			
<18.5 kg/m2	641 (5.2)	10 (4.2)	
18.5-24.9 kg/m2	8906 (72.8)	196 (82.0)	
≥25 kg/m2	2680 (21.9)	33 (13.8)	0.01*
BMI (kg/m2) median (IQR)			
All	18.8 (3.85)	18.6 (3.15)	0.05*
Girls	18.8 (3.84)	18.6 (3.37)	0.22
Boys	18.8 (3.88)	18.6 (2.67)	0.13
Weight (kg) median (IQR)			
All	47.0 (13.3)	45.2 (12.0)	0.01*
Girls	47.1 (12.9)	44.9 (13.0)	0.07
Boys	47.0 (13.6)	46.2 (11.0)	0.09
Height (cm) median (IQR)			
All	157.5 (11.0)	156.5 (11.0)	0.04*
Girls	158.0 (9.9)	156.0 (10.4)	0.04*
Boys	157.0 (11.2)	157.0 (12.6)	0.32

^a^Statistical significance defined as P ≤ 0.05 and marked with *.

The distribution of underweight, normal weight, and overweight differed significantly between the groups. Among the patients with screening-detected celiac disease, 4.2% were underweight, 82.0% were of normal weight, and 13.8% were overweight. In the group of healthy children, the proportions were 5.2%, 72.8% and 21.9%, respectively (Table 1). Using children with normal weight as a reference group, there was no association between being underweight and the risk of having undiagnosed celiac disease (OR 1.3, 95% CI 0.7–2.4). However, the risk of having celiac disease was significantly lower among overweight children (OR 0.56, 95% CI 0.4–0.8).

BMI was slightly lower among the children with screening-detected celiac disease compared to their healthy peers (median 18.6 kg/m^2^, IQR 17.1–19.8 kg/m^2^ vs. 18.8 kg/m^2^, IQR 17.2–21.1 kg/m^2^, p = 0.05).

Among the girls with screening-detected celiac disease (n = 137), 2.2% were underweight, 83.9% were of normal weight, and 13.9% were overweight compared to 6.6%, 73.8%, and 19.8% of their healthy peers, respectively (p = 0.02). The girls with screening-detected celiac disease were also significantly shorter compared to their healthy peers (median height 156.0 cm, IQR 151.6–162.0 cm vs. 158.0 cm, IQR 153.0–162.9 cm, p = 0.039). Among the boys with screening-detected celiac disease (n = 102), 6.9% were underweight, 79.4% were of normal weight, and 13.7% were overweight compared to 4.0%, 72.0%, and 24.1%, respectively, among their healthy peers (p = 0.03). Over all, the distribution of underweight, normal weight, and overweight in sex-stratified subgroups showed a similar pattern as the whole group (Table 2).

**Table 2:** Comparison of BMI between girls and boys without celiac disease (CD) and girls and boys with screening-detected celiac disease

**Groups, n (%)**	**No CD**	**CD**	**P-value**^**a**^
Girls	5932 (48.5)	137 (57.3)	
Age- and sex-adjusted BMI			
<18.5 kg/m^2^	391 (6.6)	3 (2.2)	
18.5-24.9 kg/m^2^	4376 (73.8)	115 (83.9)	
≥25 kg/m^2^	1165 (19.6)	19 (13.9)	0.02*
Boys	6295 (51.5)	102 (42.7)	
Age- and sex-adjusted BMI			
<18.5 kg/m^2^	250 (4.0)	7 (6.9)	
18.5-24.9 kg/m^2^	4530 (72.0)	81 (79.4)	
≥25 kg/m^2^	1515 (24.0)	14 (13.7)	0.03*

^a^Statistical significance defined as P ≤ 0.05 and marked with *.

## Discussion

In this large population-based celiac disease screening study, we found that children with untreated celiac disease were moderately shorter, weighed less, and had a slightly lower BMI compared to their healthy peers. Even though having undiagnosed celiac disease was not associated with being underweight, only a few were overweight and the majority of the children with screening-detected celiac disease had a normal weight.

In 2004, an American study found that children with tTG-positive screening-identified celiac disease weighed less compared to healthy children and other recent studies have found that children with celiac disease are less frequently overweight or obese and more often underweight than controls [15,17]. Some studies in patients with celiac disease have also found other factors associated with low BMI such as the extent of mucosal injury, presentation with diarrhea and female sex [12]. Studies performed in adults have shown similar results as in this study with regard to BMI [10,24].

Even if celiac disease is classically associated with malabsorption and weight loss, one must bear in mind that children with undiagnosed celiac disease can have different body compositions. The fact that the proportion of underweight children with celiac disease in our study was only 4.2% emphasizes that celiac disease should not be considered primarily as a malabsorption disorder. In addition, although celiac disease was found in this study to be distinctly less common among overweight children, being overweight does not necessarily rule out celiac disease.

In some studies, overweight has been found to be more common in boys with celiac disease than in girls [12,25]. In contrast, the same weight pattern was found to apply to both boys and girls and no indications of any gender differences in the proportion of overweight within the celiac disease group were found in the present study. In the current study, girls with celiac disease were significantly shorter than their healthy peers. This is consistent with other studies, including one study conducted in Finland that found that the screening-detected adolescent celiac disease subjects were not only shorter but one third also had nutritional abnormalities [16,26]. Even more extensive nutritional deficiencies were found in a recently conducted Dutch study in which 7.5% of the individuals with celiac disease were found to be underweight but the nutritional deficiencies were present even in obese patients [27]. This is important to keep in mind when considering the need for more active screening for the disease in order to prevent the progression of nutritional deficiencies.

The results regarding height are more inconsistent than those for weight. In some studies, adult men with celiac disease have been found to be shorter [24,28], but another study indicated that the mean adult height of patients with celiac disease was the same as that of the general population. In a subgroup analysis, reduced height was observed in the older, but not younger, birth cohorts with celiac disease [29]. Some other studies found that celiac disease diagnosed in childhood results in catch-up growth once a gluten-free diet is introduced, but still others found that men and women with celiac disease were shorter compared to controls [30-32]. Part of the differences found regarding height in the above-mentioned studies might be explained by differences in the investigated age span in the study populations. In the present study, it is likely that a majority of the girls at 12 years of age have reached the onset of puberty and that they are in the middle of their growth spurt. The onset of puberty corresponds to a biological (i.e., skeletal) age of approximately 11 years in girls and 13 years in boys. The timing of the pubertal growth spurt occurs earlier in girls and tends not to reach the same magnitude as that of boys. Girls average a peak growth velocity of 9 cm/year at age 12 and a total gain in height of 25 cm during the pubertal growth period. Boys attain an average peak growth velocity of 10.3 cm/year – which occurs about 2 years later than in girls – and gain 28 cm in height during the pubertal growth period [33-35]. This means that the majority of the girls participating in this study were in the middle of their peak height velocity when the study took place. It is possible, therefore, that untreated celiac disease gives them worse preconditions for the growth spurt or that the disease influences the onset of puberty and results in a delay in peak growth velocity. Additional epidemiologic research is needed to confirm these results, and more research needs to be done to understand the pathogenesis of short stature in patients with celiac disease.

The present investigation was based on a nationwide, contemporary study on celiac disease in 12,632 children. The screening-detected cases were ascertained by small-intestinal biopsies and HLA-DQ testing. However, the study has some limitations that merit consideration. First, the growth parameters, and thus the BMI, of the children were available only at the time of the screening. Repeated weight and height measurements following the initiation of a gluten-free diet would permit an evaluation of the effect of the diet on the nutritional status of the children. Although this is an important question that warrants further research, it is beyond the scope of this study. Furthermore, evaluating growth parameters at the time of the screening without the children knowing their diagnosis provides only a snapshot of the nutritional status in these children with untreated celiac disease compared to their healthy peers. Second, although the screening study included 12,632 children, statistical power might still constitute a limitation of the current study. There were only 239 children with celiac disease in this cohort, and only 102 were boys. The relatively low number of children with celiac disease might explain why no statistically significant differences in anthropometric measurements were found in this subgroup. However, at a population level, the growth parameters tended to be affected in the same way in boys as in girls and in the celiac disease cohort as a whole.

Overall, this study still has several strengths. To the best of our knowledge, it is the first large cross-sectional study where all the children enrolled were of the same age. The weight and height measurements were also performed according to standard procedures (as opposed to self-reported), and this makes them very reliable and comparable. Also, the celiac disease diagnosis was established using state of the art techniques including serologic markers and small-intestinal biopsies.

## Conclusions

The majority of the children with screening-detected celiac disease were of normal weight and there was no association between being underweight and the risk of having undiagnosed celiac disease. At a population level, the 12-year-old children with screening-detected celiac disease weighed less and were shorter compared to their peers without celiac disease, and this indicates a need to detect and treat celiac disease. However, at the individual level growth parameters are not reliable in predicting celiac disease. Although celiac disease is less common in the overweight subgroup, being overweight does not necessarily rule out celiac disease.

## Abbreviations

tTG: Transglutaminase; BMI: Body mass index; OR: Odds ratio; CI: Confidence interval.

## Competing interest

The authors declare no competing interests.

## Authors’ contributions

AI and AC designed the study and were responsible for the overall supervision of the study. All authors participated in the development of the study protocol and in carrying out the study, and each was responsible for one of the study sites. MP, LH and SH performed the clinical evaluations. AR performed quality control on the clinical data. FN was responsible for the database and for statistical support. MP and AM performed the data analyses and drafted the manuscript. All authors participated in the data interpretation and critical revision of the manuscript and approved its final version.

## Pre-publication history

The pre-publication history for this paper can be accessed here:

http://www.biomedcentral.com/1471-2431/14/165/prepub
